# Microarray‐based transcriptional profiling of a mouse model of autoimmune hepatitis

**DOI:** 10.1002/2211-5463.12953

**Published:** 2020-09-19

**Authors:** Yang Liu, Hao Chen, Jian‐heng Hao, Zhen‐cheng Li, Tiezheng Hou, Hui‐qin Hao

**Affiliations:** ^1^ College of Basic Medical Sciences Shanxi University of Chinese Medicine Jinzhong China; ^2^ Basic Laboratory of Integrated Traditional Chinese and Western Medicine Shanxi University of Chinese Medicine Jinzhong China

**Keywords:** autoimmune hepatitis, concanavalin A, Gene Ontology, KEGG, lncRNA, microarray

## Abstract

Long noncoding RNAs (lncRNAs) are RNA molecules longer than 200 nucleotides that do not typically code for a protein. lncRNAs have regulatory roles in many physiological processes, and their dysregulation can contribute to cancer, cardiovascular and neurodegenerative diseases, as well as the onset of autoimmune diseases, including systemic lupus erythematosus and rheumatoid arthritis. However, lncRNA expression changes in autoimmune hepatitis (AIH), a form of inflammation induced by immunological tolerance disorders, are poorly understood. Here, for the first time to our knowledge, we used microarrays to profile 1161 differentially expressed lncRNAs (DELs; 608 up‐ and 553 down‐regulated) and 11 512 differentially expressed mRNAs (DEMs; 5189 up‐ and 6323 down‐ regulated) in a concanavalin A‐induced AIH mouse model. We used quantitative real‐time PCR to confirm the expression of eight DELs and DEMs, and analyzed the coexpression relationship between them. Potential biological functions of screened DELs and DEMs were predicted with Gene Ontology and Kyoto Encyclopedia of Genes and Genomes analysis. DEL‐DEM interaction networks were also constructed. Our study revealed the roles of DELs and DEMs in the pathogenesis of AIH. We also provided potential candidate biomarkers that may have potential for future development into possible diagnostics or as a treatment for this disorder.

## Graphical abstract

Changes in long noncoding RNA expression in autoimmune hepatitis (AIH) have not been studied previously. We investigated differentially expressed long noncoding RNAs and differentially expressed mRNAs in a Con A‐induced AIH mouse model with microarray for the first time and reveal expression changes involved in the pathogenesis of AIH. These candidates might have potential to serve as potential diagnostic and therapeutic biomarkers for AIH.

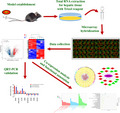

**Abbreviations**
*ACTB*: β‐actin
AIH: autoimmune hepatitis
BP: biological process
CC: cellular component
Con A: concanavalin A
DEL: differentially expressed lncRNA
DEM: differentially expressed mRNA
FC: fold change
FDR: false discovery rate
*FOXP1*: forkhead box P1
GO: Gene Ontology
*IFNG*: interferon gamma
IFN‐γ: interferon‐γ
*IL10*: interleukin‐10
*IL17RA*: interleukin‐17 receptor A
KEGG: Kyoto Encyclopedia of Genes and Genomes
lncRNA: long noncoding RNA
MAPK: mitogen‐activated protein kinase
MF: molecular function
miRNA: microRNA
PCA: principal component analysis
PCC: Pearson’s correlation coefficient
qRT‐PCR: quantitative real‐time PCR
RA: rheumatoid arthritis
*RORC*: RAR‐related orphan receptor gamma
*RUNX1*: RUNX family transcription factor 1
SLE: systemic lupus erythematosus
*TBX21*: T‐box transcription factor 21
Th: T helper
TNF‐α: tumor necrosis factor‐α

As one type of one type of progressive chronic hepatitis occurs in all ages, autoimmune hepatitis (AIH) is characterized by necrosis of hepatocytes and a rapid failure of liver function led by breaking immunological tolerance. It eventually may cause fibrosis and cirrhosis if the autoimmune process cannot be controlled [1]. However, it also starts with an episode of acute hepatitis in some cases, and these individuals are usually unresponsive to the treatment of systemic immunosuppression so that the liver transplantation is needed to avoid death [2]. Nevertheless, the etiopathogenesis of AIH was still not very clear at present. Therefore, a variety of immunological liver injury animal models, which were induced with concanavalin A (Con A) [3], S‐100 [4], lipopolysaccharide [5], as well as alcohol [6], has been established for research on AIH. Con A, isolated from *Canavalia ensiformis* seeds, can activate T cells as a mitogen by specific binding surface mannose and glucose residues. Up to now, the Con A‐induced AIH mouse model was deemed to be a convenient and reliable animal model to mimic the pathological process of AIH in human, including increased production of proinflammatory cytokines, such as tumor necrosis factor‐α (TNF‐α) and interferon‐γ (IFN‐γ), necrosis of peripheral zonal liver cells and infiltration of cytotoxic cells [7].

Long noncoding RNA (lncRNA) is a type of RNA longer than 200 nucleotides [8]. Increasing attention has been paid to the potential regulatory functions of lncRNAs participating in many physiological processes, such as transcriptional regulation in *cis* or *trans*, regulation of RNA or proteins, and organization of nuclear domains [9]. Numerous articles have also demonstrated that dysregulated expression of lncRNAs contributed to many human diseases, such as cancer, cardiovascular and neurodegenerative diseases, as well as acute liver injury and liver fibrosis [10, 11, 12, 13, 14, 15]. Furthermore, data showed that lncRNAs also play a decisive role in the onset of autoimmune diseases, including systemic lupus erythematosus (SLE) and rheumatoid arthritis (RA) [16, 17]. However, the abnormal expression profiling of lncRNAs involved in the pathogenesis of AIH has not been clarified until now.

Given the epigenetic, transcriptional and posttranscriptional regulatory effects of lncRNAs on gene expression, comprehensive understanding of the lncRNA disorders was conducive to early diagnosis, appropriate treatment and improved prognosis for patients with AIH. In this study, the characterization of these differentially expressed lncRNAs (DELs), as well as the expression profiling of differentially expressed mRNAs (DEMs), in the AIH mouse model induced by Con A was gained with microarray chip for the first time. Meanwhile, bioinformatic analyses concerning identification and biological function enrichment of the DELs were performed, not only to provide valuable information for exploring the pathogenesis mechanism of AIH, but also to establish the potential utility of lncRNAs as biomarkers or treatment targets for this disease.

## Materials and methods

### Ethics statement

Adult specific pathogen‐free male C57BL/6 mice (20–22 g, 6 weeks old) were gained from Vital River Laboratory Animal Technology Co., Ltd. (Beijing, China). National Institutes of Health *Guide for the Care and Use of Laboratory Animals* (NIH Publications No. 8023, revised 1978) was followed in this study, and all of the animal experiments were approved by the Ethics Committee of Shanxi University of Chinese Medicine (Permit Number: 2019LL41). The mice were housed under controlled temperature (21–24 °C) and humidity (40–60%), 12‐h dark/light cycle and feeding *ad libitum*, in compliance with the institutional rules of animal experimentation approved by the Experimental Animal Ethics Committee of Shanxi University of Chinese Medicine.

### Reagents and chemicals

Con A was bought from Solarbio Science & Technology Co., Ltd. (Beijing, China), batch number: C8110; chloral hydrate, TRIzol Reagent and 2× SYBR Abstart Master Mix was purchased from Sangon Biotech Co., Ltd. (Shanghai, China), batch number: A600288, B610409 and B110032.

### Animal experiment and sample collection

Con A (15 mg·kg^−1^, dissolved in pyrogen‐free saline) was administrated to the mice in the model group (*n* = 4) via tail‐vein injection. The sham group mice (*n* = 4) were given pyrogen‐free saline alone with the same method. All mice were anesthetized lethally at 8 h after the administration of Con A or pyrogen‐free saline. Hepatic tissues of each mouse were harvested by surgery under low‐temperature and sterile conditions, and homogenized immediately for extracting total RNA with TRIzol Reagent. The extracted RNA was quantified by the NanoDrop ND‐2000 (Thermo Scientific, Madison, WI, USA), and Agilent Bioanalyzer 2100 (Agilent Technologies, Boston, MA, USA) was used to assess the integrity of total RNA.

### Microarray hybridization and data analyses

OE biotech Mouse Microarray (version 2018; OE Biotech. Co., Ltd., Shanghai, China), which was designed for the global profiling of mouse protein coding transcripts and lncRNAs according to GRCm38.p6 (data update in October 3, 2017), was used in this experiment. It approximately contained 12 461 lncRNAs (constructed based on RNA‐sequencing databases) and 20 052 coding transcripts (each transcript was represented by a specific exon or splice junction probe). For controlling the hybridization quality, positive probes for housekeeping genes and negative probes were also printed onto the microarray. The sample labeling, microarray hybridization and washing were performed according to the manufacturer's standard protocols. feature extraction software (version 10.7.1.1; Agilent Technologies) was used to analyze array images to get raw data. genespring (version 14.8; Agilent Technologies) was used to finish the basic analysis with the raw data. To begin with, the raw data were normalized with the quantile algorithm. The standardized data were filtered for subsequent analysis provided the conditions that at least 75% of the samples labeled as ‘detected’ were met. Correlation analysis between samples was implemented with Pearson’s correlation and principal component analysis (PCA) to evaluate the reproducibility of the data. DELs and DEMs were then identified through fold change (FC), as well as *P* value calculated with *t*‐test. The thresholds set for up‐ and down‐regulated expression genes were |log_2_FC| > 1 and *P* < 0.05. Hierarchical clustering analysis was performed to reveal the clustering relationships between these differentially expressed genes.

### Quantitative real‐time PCR

To validate the microarray data, we selected eight DELs (four up‐ and four down‐regulated) from all aberrantly expressed lncRNAs for quantitative real‐time PCR (qRT‐PCR) amplification with one‐step SYBR Green assay in a GeneAmp PCR System 9700 (Applied Biosystems, Madison, WI, USA). At the same time, eight DEMs (six up‐ and two down‐regulated) were also singled out for qRT‐PCR amplification. Features of the chosen DELs and DEMs were presented in Table 1. The primers were designed in the laboratory and synthesized by Generay Biotech Co., Ltd. (Shanghai, China) based on the sequences obtained from the National Center of Biotechnology Information database; sequences of the primers were listed in Table 2. The expression levels of DELs and DEMs were normalized to *β‐actin* (*ACTB*) and were calculated using the 2^−ΔΔCt^ method [18].

**Table 1:** Features of the DELs and DEMs selected for qRT‐PCR validation.

Accession	Gene symbol	FC	*P* value	Regulation
XR_001782679.1	*GM36043*	25.80	1.86e−5	Up
NR_137283.1	*G530011O06RIK*	10.62	2.74e−5	Up
XR_879376.2	*GM31718*	10.06	6.94e−4	Up
NR_033590.1	*GM8096*	9.37	1.21e−9	Up
NR_040346.1	*1810019D21RIK*	−4.86	1.99e−3	Down
XR_380679.2	*GM32468*	−3.25	2.62e−3	Down
XR_872832.2	*GM40444*	−2.85	1.77e−3	Down
XR_001778054.1	*MYPOPOS*	−2.68	1.14e−3	Down
NM_008337.4	*IFNG*	21.63	4.34e−7	Up
NM_008359.2	*IL17RA*	11.77	3.68e−5	Up
NM_009821.3	*RUNX1*	7.38	9.25e−4	Up
XM_006507388.1	*IL4RA*	4.69	2.97e−6	Up
NM_010548.2	*IL10*	3.74	5.64e−3	Up
NM_019507.2	*TBX21*	3.45	2.46e−5	Up
XM_006501163.2	*RORC*	−3.23	1.76e−4	Down
XM_006505316.3	*FOXP1*	−2.18	1.68e−3	Down

**Table 2:** The sequences of primers used in qRT‐PCR experiments.

Gene symbol	Forward primer (5ʹ–3ʹ)	Reverse primer (5ʹ–3ʹ)	Product length (bp)	*T*_m_ (°C)
*ACTB*	GGCTCCTAGCACCATGAAGA	AGCTCAGTAACAGTCCGCC	187	60
*GM36043*	AGTAGCAGCATAGATTGAAGC	AAGATAATGCGAGGTGCC	86	60
*G530011O06RIK*	ACTCTTCTGTCAAGGGAACT	AATCCTGGTCATGTACCCAA	128	60
*GM31718*	TGCATGATGCTTAGAAGGCATA	CACGTCTGTCAGTGAAGAATAG	102	60
*GM8096*	ATTGGAGGCTTTCCAGTTC	TAGGTCACAGTGAGAGGGTT	91	60
*1810019D21RIK*	GGGAGATCTTGCTGACTGTG	TGAATGGAGCCGCTCAAT	122	60
*GM32468*	AGTCCTATGGCCTAGAGC	TTCTTCCTCCATGTCTGGTTG	106	60
*GM40444*	AATTGTAGCACTTAAGGCTGAG	GTTGGTATGGTATCCTTGGTC	106	60
*MYPOPOS*	ACTCCATCATCCCTCATTCT	ACGACGACTCCTTAGGTG	90	60
*IFNG*	CTGTTTCTGGCTGTTACTGC	TCTTCCACATCTATGCCACT	91	60
*IL17RA*	CGTGGCATAACAGGAGAAC	CCCTGGAGCTACTTTAAACAAC	98	60
*TBX21*	TCGTGGAGGTGAATGATGG	TGATCTCTGCGTTCTGGTAG	117	60
*IL10*	GACAACATACTGCTAACCGAC	TCCTTGATTTCTGGGCCAT	137	60
*RUNX1*	GGTACTGTTGTTACAGTGGAG	CATGTATGTCAGAACCAGCG	85	60
*IL4RA*	TCCTCCGCTCAGTTGTAG	TGGCTTCTGGTCATATGTGG	86	60
*RORC*	CTTTCCCTTTCTGCACTCTATG	CTTGCTGCTGTTGTCCTAC	104	60
*FOXP1*	TCTTTAATCAGGCAGGCCAT	TGCGTCGGAAGTAAGCAA	100	60

### Coexpression analysis of DELs and DMEs

Based on the chromosome's location of these DELs and DEMs, the interactions between DELs and DEMs were identified in accordance with the normalized signal intensities of gene expression levels. In this study, the Pearson’s correlation coefficient (PCC) was used to calculate the significant associations between DELs and DEMs, and lncRNA‐mRNA coexpression pairs with |PCC| ≥ 0.80 and *P* ≤ 0.05 were selected. The coexpression networks of the eight validated DELs were formulated with cytoscape software (version 3.7.2, http://cytoscape.org/).

### Function prediction of DELs and DMEs

Gene Ontology (GO) enrichment analysis [19] was conducted with the method of hypergeometric distribution algorithm [20] by using the r Programming Language software [21] to have a deep insight into the characteristics of the eight validated DELs (in line with the DEL‐DME coexpression analysis results) and all DEMs in terms of cellular components (CCs), molecular functions (MFs) and biological processes (BPs). The false discovery rate (FDR), applied to the multiple testing corrections of raw *P* value, was set as the cutoff for selecting significantly enriched functional GO terms (FDR ≤ 0.05). The underlying biological functions of these eight chosen DELs and all DEMs were predicted with Kyoto Encyclopedia of Genes and Genomes (KEGG) enrichment analysis (Release 85.0, January 1, 2018; https://www.genome.jp/kegg/). The significance of each differentially expressed gene annotated in the pathways was also calculated with the method of hypergeometric distribution algorithm [20] by using the r Programming Language software [21]. The recommended FDR ≤ 0.05 was the threshold for genes that were eligible to be finally annotated in one pathway, and lower FDR values represented higher correlation between KEGG pathways and target genes.

### Statistical analysis

All data were statistically analyzed by independent‐samples *t*‐test to identify the DELs and DEMs with Statistic Package for Social Science (spss) 25.0 software (SPSS Inc., Chicago, IL, USA). Statistical significance was considered as *P* < 0.05.

## Results

### Identification

**Fig. 1:**
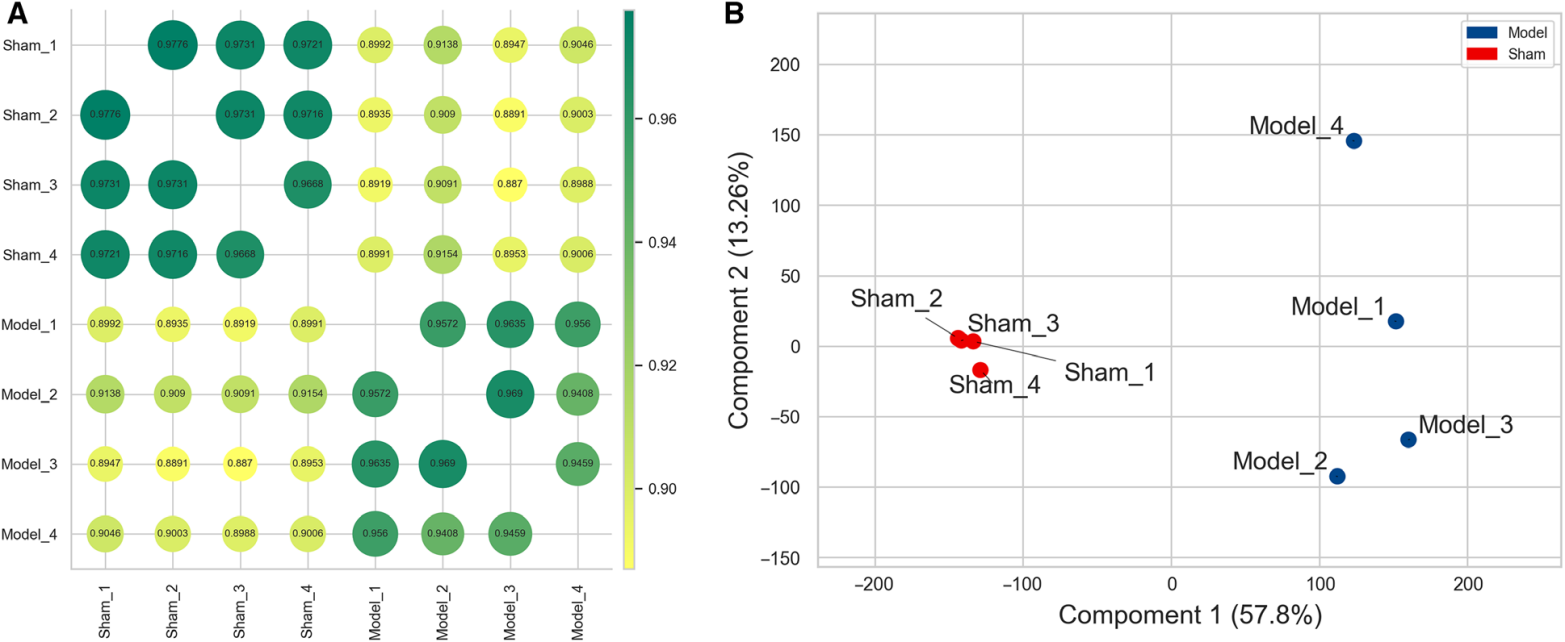
Correlation analysis between different samples. (A) Pearson’s correlation analysis. Each dot represented the correlation between different samples, and the decimal in the dot was the coefficient of association. The larger the number, the greener and bigger the dot, which meant the higher the relevance. (B) Results of PCA. The red and blue dots signified the individuals in model and sham groups, respectively. The shorter the distance between samples in the same group, the more significant was the separation between samples in different groups, which indicated the more the experimental reliability and rationality of sample selection.

As shown in Fig. 1A, each dot represented the correlation between different samples, and the greener the dot, the higher the correlation. The normalized data of each sample in the sham group were with a very high correlation, and the same for the model group. According to the results of PCA in Fig. 1B, we also found that the distance between samples in the same group was relatively close, and the samples in different groups were relatively discrete, that is, individuals in the model group flocked together and separated significantly from the sham group. It was indicated that experimental reliability and rationality of sample selection satisfied the conditions for further analysis, and the reproducibility of the data was revealed.

**Fig. 2:**
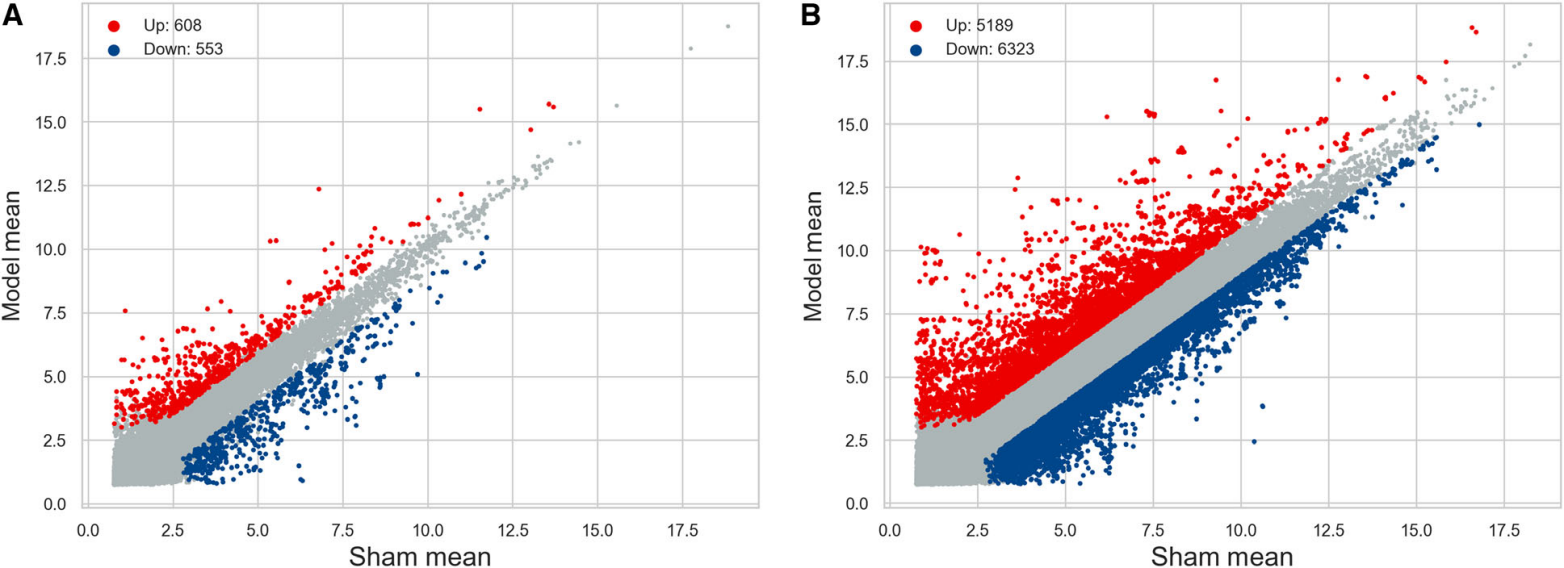
Scatter analysis of the (B) DEMs and (A) DELs. Scatter diagrams were used to distinguish the DEMs and DELs with the threshold of *P* < 0.05 and |log_2_FC| > 1. The horizontal lines and vertical lines represented the expression levels of genes in the sham group and the AIH model group, respectively. The red and blue points reflected the up‐ and down‐regulated genes, and the gray points represented genes expressed that did not meet the threshold.

**Fig. 3:**
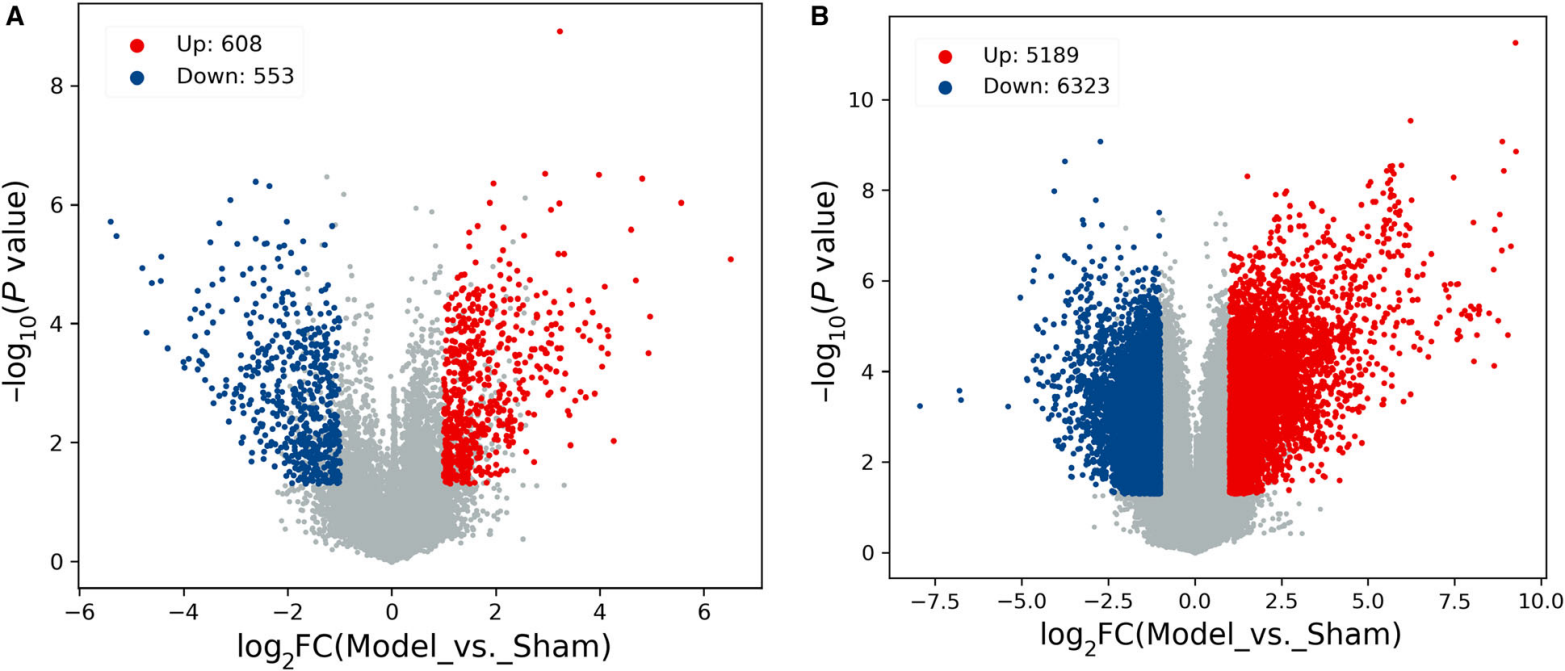
Volcano plot filtering of the (B) DEMs and (A) DELs. The red and blue points highlighted up‐ and down‐regulated genes, and the gray points represented nondifferentially expressed genes. Higher absolute value of abscissa denoted the greater FC between the two groups. The higher the value of ordinate was, the more the differential expression of genes was reliable.

**Fig. 4:**
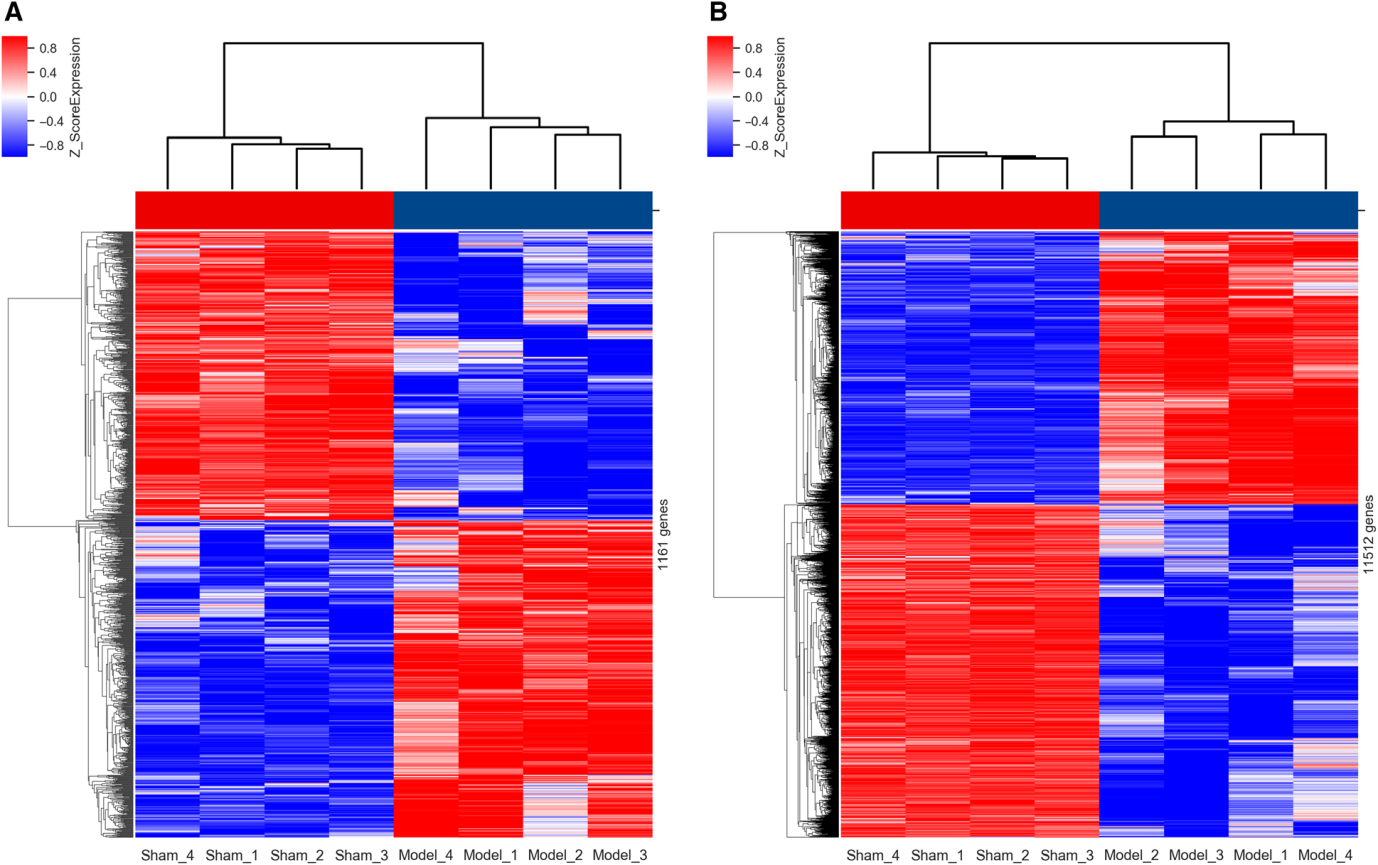
Hierarchical clustering analysis of the (B) DEMs and (C) DELs. The red and blue shades represented the expression levels above and below the relative expression among all samples, respectively. Sham 1–4: mouse in AIH sham group; Model 1–4: mouse in model group.

With the threshold of *P* < 0.05 and |log_2_FC| > 1, 1161 DELs (608 up‐ and 553 down‐regulated) and 11 512 DEMs (5189 up‐ and 6323 down‐regulated) were identified in the Con A‐induced AIH model group compared with the sham group. For making screened results intelligible, the identification data were visualized by scatterplot and volcano plot. As shown in Figs 2 and 3, the filtered‐out up‐ (red points) and down‐regulated genes (blue points) were distinctly set apart from the genes that did not meet the threshold (gray points), which not only manifested that the microarray‐based screening results were reliable, but also gave us some clues to select target genes for further studies. The top 10 (according to the descending order of |FC|) up‐ and down‐regulated DELs and DEMs were itemized in Tables S1 and S2. *Gm39247* (91.1‐fold) and *Gm41999* (42.4‐fold) were the most significantly up‐ and down‐regulated DELs, whereas *Olr1* [musculus oxidized low‐density lipoprotein (lectin‐like) receptor 1; 615.6‐fold] and *cytochrome P450, family 7, subfamily a, polypeptide 1* (244.7‐fold) were the most significantly up‐ and down‐regulated DEMs. In addition, the clustering relationships between these differentially expressed genes were revealed by hierarchical clustering analysis, and all mice in the model group were clustered together and separated from the individuals in the sham group, as exhibited with hierarchical clustering heatmaps in Fig. 4. Thus, the significance of the microarray‐based screening results for the study on the pathogenesis of AIH was reconfirmed by the hierarchical clustering analysis.

### qRT‐PCR validation

**Fig. 5:**
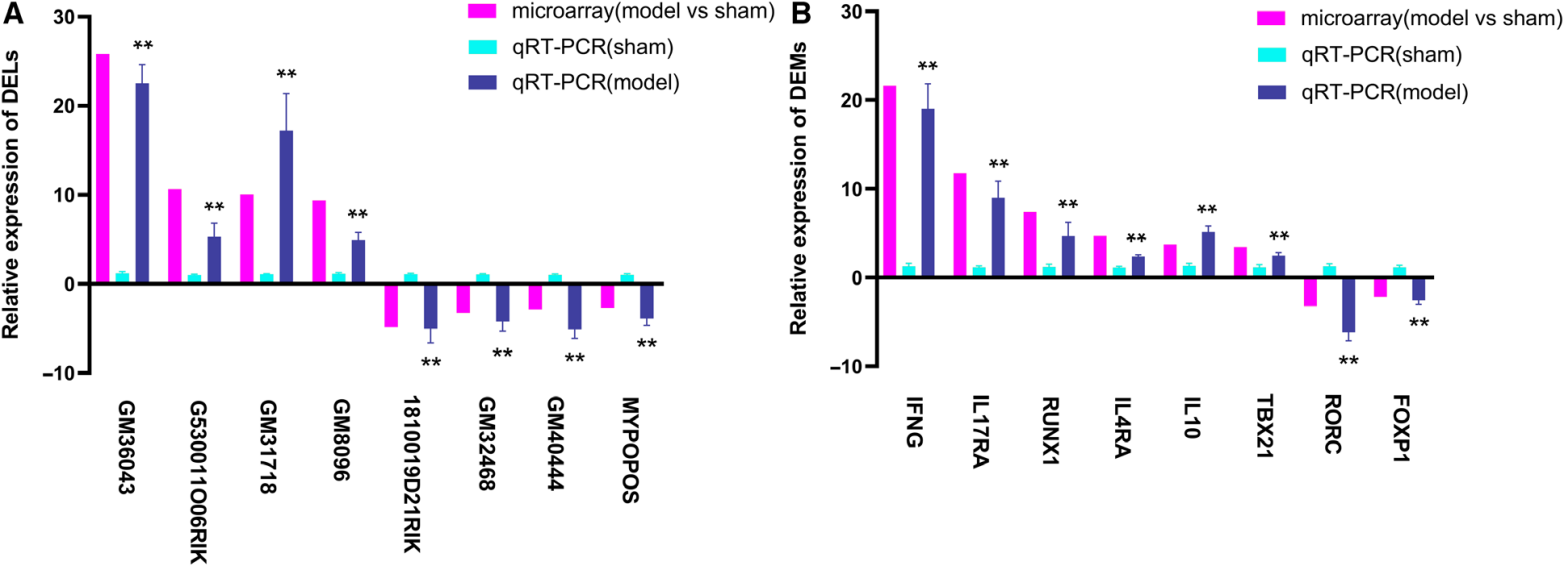
Relative expression of the selected DEMs and DELs. *y* axis signified the relative expression level of the chosen genes in the model group (*n* = 4) compared with that in the sham group (*n* = 4), and the negative values indicated expression of the gene in the model group was down‐regulated. Magenta column represented the results of microarray (the data were normalized to the negative probe), while cyan and blue columns reflected the findings in sham and model groups with qRT‐PCR (the data were normalized to *ACTB*). The data were shown with the mean ± SD (error bars) and statistically analyzed by Student's *t*‐test. *P* < 0.05 was considered statistically significant (**P < 0.05). (A) DELs: *GM36043* (22.53 ± 2.08), *GM31718* (17.21 ± 4.14), *G530011O06RIK* (5.27 ± 1.56), *GM8096* (4.94 ± 0.84), *GM40444* (−5.08 ± 1.03), *1810019D21RIK* (−5.05 ± 1.59), *GM32468* (−4.22 ± 1.07) and *MYPOPOS* (−3.89 ± 0.77). (B) DEMs: *IFNG* (19.03 ± 2.82), *IL17RA* (9.01 ± 1.85), *IL10* (5.13 ± 0.70), *RUNX1* (4.68 ± 1.55), *TBX21* (2.46 ± 0.36), *IL4RA* (2.37 ± 0.18), *RORC* (−6.13 ± 1.0) and *FOXP1* (−2.57 ± 0.46).

Compared with the sham group, the expression differences of these selected eight DELs and DEMs in the model group detected by qRT‐PCR assay were statistically significant (*P* < 0.05). As laid out in Fig. 5, the expression level of *GM36043* (22.53‐fold) was the most elevated, followed by *GM31718* (17.21‐fold), *G530011O06RIK* (5.27‐fold) and *GM8096* (4.94‐fold), whereas *GM40444* (5.08‐fold) was the most down‐regulated DEL, and the next were *1810019D21RIK* (5.05‐fold), *GM32468* (4.22‐fold) and *MYPOPOS* (3.89‐fold) in turn. For these up‐regulated DEMs, the FCs in descending order were 19.03‐fold for interferon gamma (*IFNG*), 9.01‐fold for interleukin‐17 receptor A (*IL17RA*), 5.13‐fold for interleukin‐10 (*IL10*), 4.68‐fold for RUNX family transcription factor 1 (*RUNX1*), 2.46‐fold for T‐box transcription factor 21 (*TBX21*) and 2.37‐fold for *IL4RA*. The expression level of RAR‐related orphan receptor gamma (*RORC*) (6.13‐fold) was the most declined, while the second one was forkhead box P1 (*FOXP1*) (2.57‐fold). For both of these chosen DELs and DEMs, the qRT‐PCR results were in accordance with the microarray analyses, which provided truthful evidence to testify to the credibility of microarray‐based screening results.

### Coexpression analysis

**Fig. 6:**
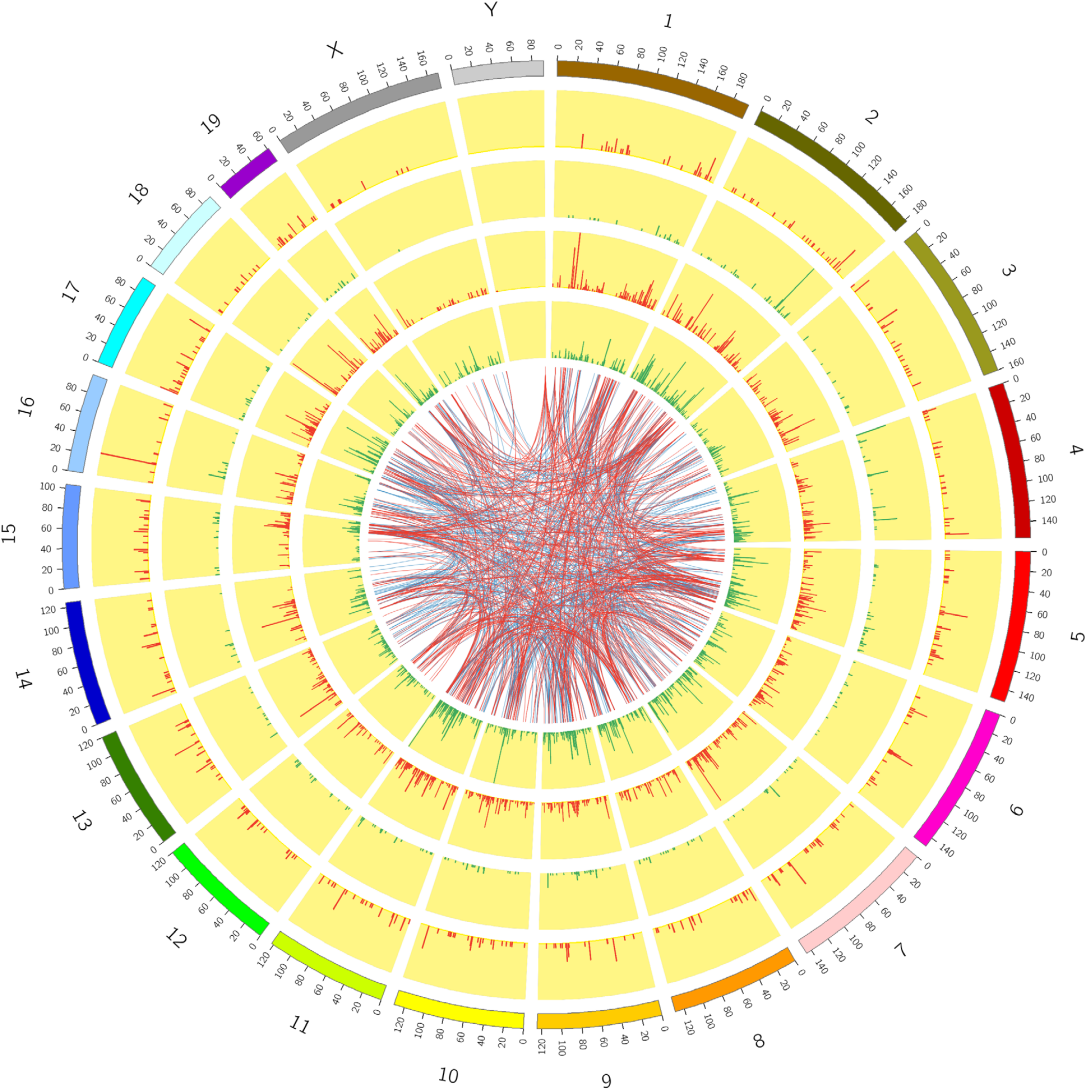
DEL‐DEM coexpression analysis. Circos plot was used to visualize the chromosome's location and interactions between the coexpressed DEMs and DELs. The outermost circle was the autosomal distribution of mouse. The second and third circles showed the distribution of DEMs on chromosomes; red and green lines indicated the up‐ and down‐regulated genes. The higher the column, the greater the was the number of DEMs in this region. The fourth and fifth circles showed the distribution of DELs on chromosomes, with the same expression form as DEMs. The internal connection indicated the corresponding relationship between the top 500 coexpressed DEMs and DELs.

**Fig. 7:**
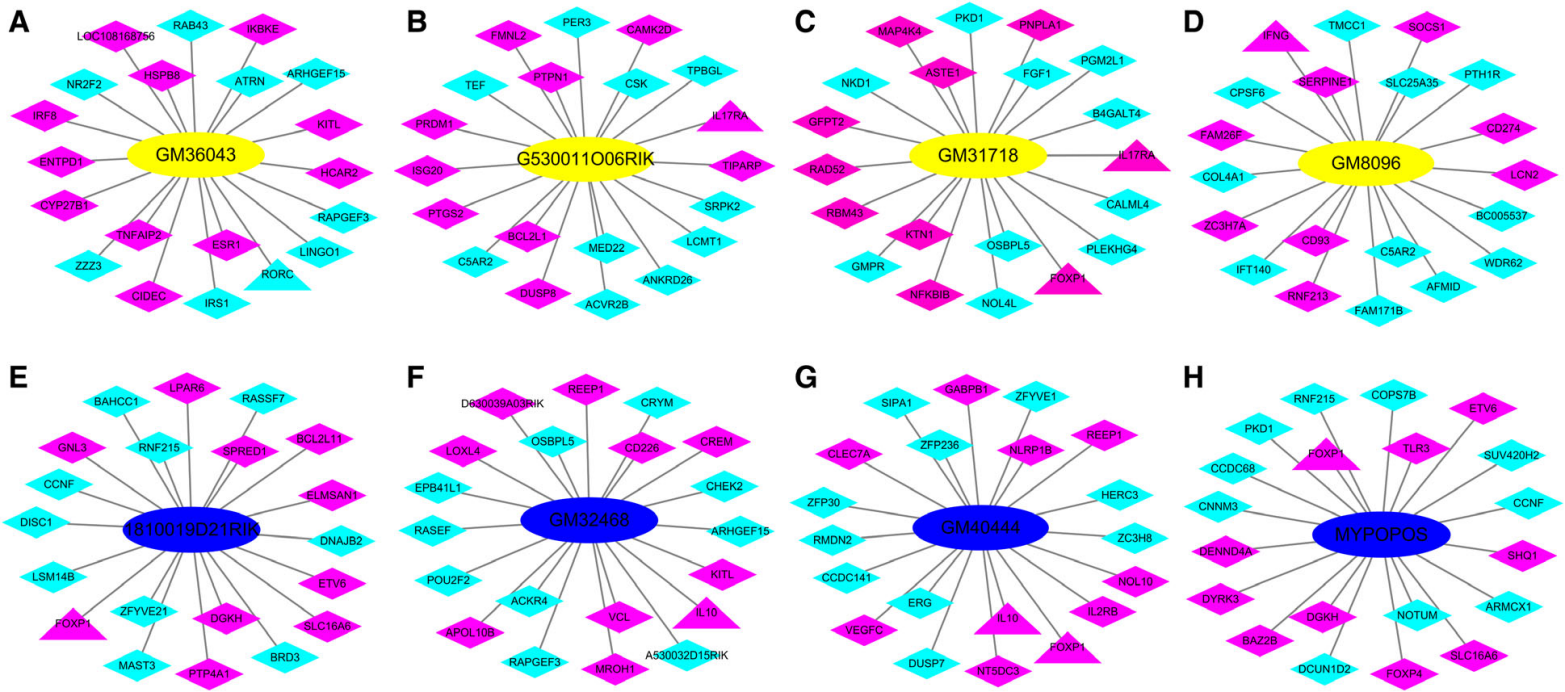
DEL‐DEM coexpression networks of the eight chosen DELs. Yellow and blue ellipse nodes indicated the up‐ and down‐regulated DELs; magenta and cyan diamond nodes represented the up‐ and down‐regulated DEMs, respectively. Triangle nodes represented the DEMs that have been validated by qRT‐PCR. (A) *GM36043*. (B) *G530011O06RIK*. (C) *GM31718*. (D) *GM8096*. (E) *1810019D21RIK*. (F) *GM32468*. (G) *GM40444*. (H) *MYPOPOS*.

The locations of these DELs and DEMs on chromosomes were displayed in Fig. 6. The DELs and DEMs distributed on all chromosomes (including 1–19, X and Y). Resembling the location of differentially expressed microRNAs (miRNAs) in the same model (data not shown), the numbers of both DELs and DEMs expressed on chromosome 2 were larger than that expressed on the other chromosomes. It was indicated that the abnormity of chromosome 2 was closely bound up with the development of AIH. On this basis, the intricate coexpression relationship between these screened DELs and DEMs was analyzed according to the set threshold, and the top 500 (in ascending sort order of *P* value) DEL‐DEM coexpression pairs were also visualized with Circos plots in Fig. 6. The top 20 (in terms of *P* value with ascending sort order) DEL‐DEM coexpression pairs were presented in Table S3. The eight validated DELs coexpressed separately with 24, 59, 9, 74, 160, 204, 4 and 45 DMEs with the threshold of |PCC| ≥ 0.99 (*GM32468* with threshold of |PCC| ≥ 0.95). The coexpression networks of these DELs with the top 10 (in descending order of the |PCC|) negatively or positively coexpressed DEMs were constructed in Fig. 7, respectively. The top three negatively and positively coexpressed DMEs of the eight verified DELs were listed in Table S4. It was denoted that comprehending coexpression pairs is helpful not merely to elucidate the pathogenesis of AIH, but also to provide new ideas and means for treating this disorder.

### Functional annotation

**Fig. 8:**
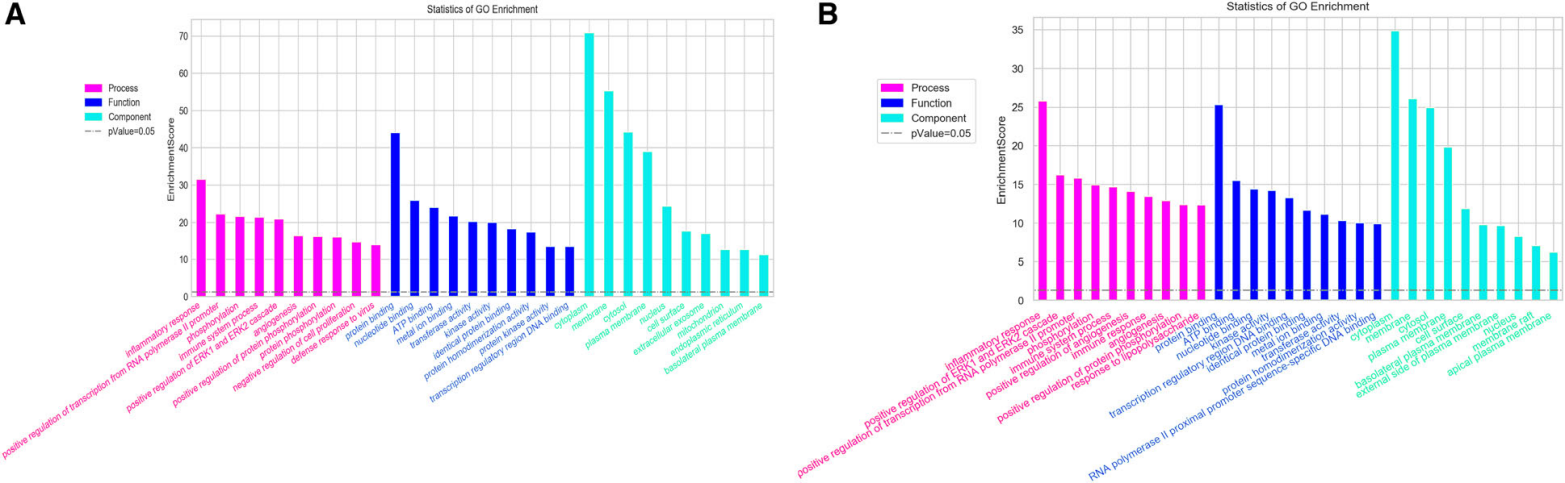
GO enrichment analysis. GO enrichment analysis of differentially expressed genes was performed based on hypergeometric distribution using the r Programming Language software. Top 10 terms of different GO categories were listed on the horizontal line. The magenta, blue and cyan column graphs represented the BPs, MFs and CCs, respectively. The vertical line corresponded to the enrichment score [−log10(*P*)]. (A) All DEMs. (B) The validated DELs.

Our results showed that 10 970 of 11 512 DEMs were annotated in 685 GO terms of different categories with FDR ≤ 0.05 (468 in BP, 91 in CC and 126 in MF). All of the DEMs were mostly responsive to ‘inflammatory response’ (GO:0006954) (BP), ‘protein binding’ (MF) and enriched in ‘cytoplasm’ (CC; in Fig. 8A), which emphasized the role of inflammatory response in the progress of hepatic lesion. Furthermore, GO enrichment analysis of each validated DEL was exhibited in Fig. 8B, according to the coexpression DEMs annotated in the categories of CC, MF and BP. There were 3388 of 3497 coexpression genes annotated in the 463 GO terms (323 in BP, 51 in CC and 89 in MF) with the threshold of FDR ≤ 0.05 for these eight validated DELs. Notably, the coexpressed genes of these DELs were also significantly involved in the BP category of ‘inflammatory response,’ which gave us some new insights about how this BP was closely involved in the occurrence and development of this immune‐mediated disorder.

**Fig. 9:**
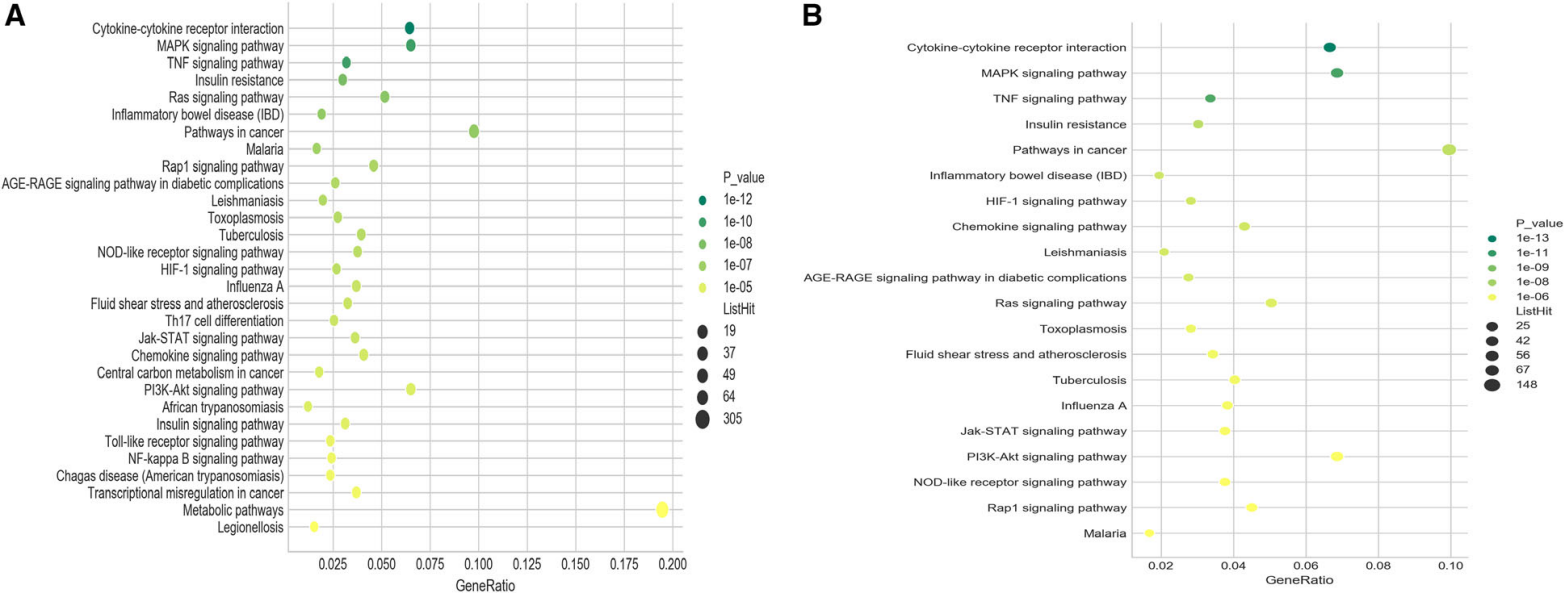
KEGG enrichment analysis. KEGG pathway enrichment analysis of differentially expressed genes was performed based on hypergeometric distribution using the r Programming Language software. The horizontal line corresponded to degree of enrichment (gene ratio), and the vertical line represented the enriched pathways. The larger the point, the more genes that were enriched in the pathway, whereas the greener the point, the higher the enrichment significance. (A) Top 30 pathways in which all DEMs were enriched. (B) Top 20 pathways connected with the eight validated DELs.

Subsequent KEGG enrichment analysis presented that 1567 of 11 512 DEMs were significantly enriched in 108 signaling pathways with FDR ≤ 0.05, and the top three significantly enriched pathways (in ascending sort order of FDR value) were ‘cytokine‐cytokine receptor interaction’ signaling pathway (KEGG PATHWAY: mmu04060), ‘mitogen‐activated protein kinase (MAPK)’ signaling pathway (KEGG PATHWAY: mmu04010) and ‘TNF’ signaling pathway (KEGG PATHWAY: mmu04068) (Fig. 9A). These results suggested that abnormalities in these signaling pathways were the key to this disease. In addition, our findings also revealed 1487 of 3497 coexpressed DEMs were significantly enriched in 99 signaling pathways for these eight chosen DELs with FDR ≤ 0.05. The top 20 signaling pathways (in ascending sort order of FDR value) in which these coexpressed DEMs enriched were shown in Fig. 9B, and the top three significantly enriched pathways (in ascending sort order of FDR value) for these validated DELs were also ‘cytokine‐cytokine receptor interaction’, ‘MAPK’ and ‘TNF’ signaling pathways. These results provided a better understanding of the underlying regulatory function of these DELs and how these DELs possibly participated in the onset of AIH.

## Discussion

AIH, a type of immune‐mediated injury targeting liver parenchyma, has a high prevalence in Europe and the United States [22]. However, an efficient cure for AIH is presently lacking. Lack of well‐established animal models, as well as novel biomarkers, was detrimental to the scientific research on AIH. So far, although diversified animal models for AIH have been used to mimic the process of liver injury in human, the Con A‐induced liver injury model was still regarded as the best experimental model in this research field. The advantages of this model were convenient, inexpensive and repeatable to investigate the macrophage and T‐cell‐dependent liver injury. Similar to in human patients with AIH, the typical peripheral zonal necrosis in liver and increased production of proinflammatory cytokines, including TNF‐α and IFN‐γ, were also found in this model.

Despite not being translated into proteins, accumulating evidence in recent years supported that lncRNAs were critical regulators in many physiological and pathological processes by interacting with DNA, RNA and protein [23]. For example, lncRNAs could increase the expression of mRNAs by acting as competing endogenous RNA to competitively bind to miRNA via the base complementary. For many autoimmune diseases (such as RA, SLE, psoriasis, autoimmune thyroid disease and Crohn's disease), dysregulation of lncRNAs correlated with the abnormal development of immune cells (e.g., dendritic cells, granulocytic and T cells) and expression of proinflammatory cytokines (including IFN‐γ) [24, 25, 26, 27, 28]. Nevertheless, whether some aberrantly expressed lncRNAs take part in the etiopathogenesis of AIH has rarely been reported.

For the reasons mentioned earlier, we revealed the expression pattern of DELs and DEMs in the Con A‐induced AIH mouse model with the forefront technology of microarray chip for the first time. Compared with the healthy shams, 1161 DELs (608 up‐ and 553 down‐regulated) and 11 512 DEMs (5189 up‐ and 6323 down‐ regulated) were identified in this AIH model. As shown in Figs 1 and 2, these screened DELs and DEMs (blue points and red points) were separated distinctly from the genes not reaching the set threshold (gray points), which reflected the exact distribution trend of these differentially expressed genes. lncRNAs and mRNAs that exceed the threshold were somehow related to the onset of AIH, and the more the gene is far away from the threshold, the more it is necessary to explore its biological function involved in the pathogenesis of this disease in‐depth. Meanwhile, the hierarchical clustering analysis also revealed the discriminatory ability of these DELs and DEMs, because it was capable of stratifying the AIH models from the sham individuals according to the results displayed with heatmaps in Fig. 3. These results evinced that these filtered DELs and DEMs have the potential to play a pivotal role in the process of AIH as well.

To validate the credibility of microarray‐based screening results, we picked up eight DELs and eight DEMs for qRT‐PCR verification. The qRT‐PCR results of the chosen DELs and DEMs were in accordance with the results obtained from the microarray analysis (see Fig. 4). It was signified that our microarray‐based screening data were reliable and available for follow‐up studies (such as coexpression analysis and function prediction), as well as brought up another hint that the relationship between the eight validated DELs and AIH is worth studying in the future.

One of the most important biological features for lncRNAs was to regulate the expression or function of target genes. For the sake of studying the coexpression relationships between DELs and DEMs, the analysis of location on chromosome for these DELs and DEMs was performed and exhibited with Circos plot in Fig. 5. All chromosomes (consisting of 1–19, X and Y) were affected in the development of AIH, and chromosome 2 was the most severely affected one. Combined with the results of location of differentially expressed miRNAs in the same model (data not shown), it was suggested that there were some uncleared associations between onset of AIH and chromosome 2. A good many of the DEL‐DEM coexpression pairs were found in this article, and the top 500 (in ascending sort order of *P* value) were also visualized with Circos plot in Fig. 5. A penetrating investigation on the complicated regulatory relationships between DELs and DEMs will contribute immensely to exploring the pathogenesis of AIH. Then the coexpression networks of the eight validated DELs with the top 10 negatively and positively coexpressed DMEs were also constructed and set out in Fig. 6. The results offered some new clues for how to further explore the regulating mechanism of the eight validated DELs. Unfortunately, there were nearly no correlative reports on the biological function of the eight validated DELs until now. Nevertheless, we found most of them to be highly expressed in immune organs, such as thymus and spleen, with the sort of reads with per kilobase per million reads placed according to the National Center for Biotechnology Information gene database (https://www.ncbi.nlm.nih.gov/gene), which hinted that these DELs are likely to take some part in the BPs of certain immune responses.

To explore the potential biological function of differentially expressed genes in depth, we conducted bioinformatics analysis (consisting of GO and KEGG enrichment analysis). GO analysis results showed that all DEMs and the coexpressed DEMs of the eight validated DELs were both significantly involved in the BP category of ‘inflammatory response’ (GO:0006954), a GO term defined as ‘the immediate defensive reaction to infection or injury caused by chemical or physical agents’ (see Fig. 7). As known from the published reports on AIH, disturbance of immunological tolerance was the main reason for the necrosis of liver cells, and increased production of proinflammatory cytokines was one of the typical pathological features in this disease. The GO results presented here emphasized the feature of immune dysfunction in AIH once again and revealed the regulation feature of these DELs in the process of inflammation.

For the KEGG enrichment analysis, our study also demonstrated the key signaling pathways that were possibly modulated by the eight selected DELs, including ‘cytokine‐cytokine receptor interaction’, ‘MAPK’ and ‘TNF’ signaling pathway, which resembled the results of all DEMs. Interestingly, it has been reported that the abnormal changes of ‘MAPK’ and ‘TNF’ signaling pathways played essential roles in the pathophysiological processes of inflammation in liver, and these two signaling pathways were deemed to be served as the key pathways as therapeutic targets for AIH [29, 30]. Although the exact relationship between ‘cytokine‐cytokine receptor interaction’ signaling pathway and AIH has not been identified, studies have reported that this pathway was an explicit biological target for the diagnosis and treatment of RA [31]. As another autoimmune disorder common to humans, RA shares some molecular pathogenesis with AIH (e.g., impaired programmed death‐1 and its ligands), and 2–4% of patients with RA were diagnosed with AIH [32, 33]. Therefore, the ‘cytokine‐cytokine receptor interaction’ signaling pathway is also likely to play an important role in the pathogenetic process of AIH, and a thorough understanding of this pathway’s effects on the abnormal immune response in this disease is required. Our findings presented here not only corroborated the specific role of these pathways involved in the pathogenesis of AIH, but also was helpful to gain a deeper understanding of why the activity of these pathways changed in this disease. Moreover, the other enriched pathways that have not yet been proved to be correlated with the development of AIH were needed to lucubrate whether they can be the latent mechanisms and serve as the promising candidate therapeutic targets for this disease.

In addition, significant abnormal expressions of the eight chosen DEMs in the model group, including *IFNG*, *TBX21*, *IL17RA, IL4RA, IL10, RUNX1*, *RORC* and *FOXP1*, were also detected with qRT‐PCR assay, which indicated that these genes are closely related to the incidence of AIH. In light of the existing literature, IFN‐γ (encoded by *IFNG*) and T‐bet (encoded by *TBX21*) have been regarded as the most essential proinflammatory cytokine for the homing of autoreactive T lymphocytes to liver and the crucial transcription factor for T helper (Th) 1 cells differentiation in the development of AIH [34, 35, 36, 37]. Except for the increased expression of *IFNG* and *TBX21* found in the Con A‐induced AIH model, we also found *IFNG* and *TBX21* were incorporated into the coexpression networks of *GM8096* and *GM31718* (two of the validated DELs), respectively. It was pointed out that the expressions of *IFNG* and *TBX21* were probably modulated by lncRNAs *GM8096* and *GM31718*, which shed some light on the question of how *IFNG* and *TBX21* were involved in the pathogenesis of AIH.

It was well known that activated Th17 cells, the major source of IL‐17, take part in the pathogenetic process of many autoimmune diseases, including psoriatic arthritis, experimental autoimmune encephalomyelitis and AIH [38, 39, 40]. RORγ, the transcription factor that plays a crucial part in the differentiation of Th17, is encoded by *RORC* [41]. Studies have shown that modulated expression of RORγ selectively was an effective method in treating Th17‐associated autoimmune diseases [42]. As one member of the IL‐17 receptor family and encoded by *IL17RA*, IL‐17RA was capable of activating several downstream inflammatory pathways, such as the ‘nuclear factor kappa B’ signaling pathway and ‘MAPKs’ signaling pathway [43]. Apart from increased expression of *IL17RA* and decreased expression of *RORC* being found in the Con A‐induced AIH model, our findings also revealed that *IL17RA* was coexpressed with lncRNAs *G530011O06RIK* and *GM31718*, whereas *RORC* was exhibited in the network of lncRNA *GM36043* (in Fig. 6). It is implied that the aberrant expression of *IL17RA* and *RORC* had something to do with these DELs, and a new understanding of the biological function of *IL17RA* was sparked for it is a potential hub target gene regulated by different lncRNAs. In addition, expression change of *RORC* detected by both microarray chips and qRT‐PCR in our study was not consistent with the findings in other studies focused on the late stage of AIH [44]. It is indicated that the mechanisms of *RORC* that participated in the different phase of AIH are divers, and the exact mechanisms needed to be explored deeply.

Some recent studies confirmed that *IL10* (encoding IL‐10) and *FOXP1* (encoding Foxp1) participate in maintaining the balance of the immune system by regulating the methylation frequency of *FOXP3* and formation of Foxp1/Foxp3 heterodimer [45, 46, 47], whereas reduction in expression and deficiencies in function of Foxp3 are hypothesized to contribute to the pathogenesis of AIH [48]. The aberrant expression of *IL10* and *FOXP1* was associated with various immune‐mediated inflammatory diseases, including RA, SLE and inflammatory bowel disease [49, 50, 51, 52, 53], and the importance of IL‐10 in immune regulation for AIH has been recognized [54, 55]. As rendered in our article, the differential expressions of *IL10* and *FOXP1* were detected, and we also found that *IL10* coexpressed with *GM32468* and *GM40444*, and *FOXP1* coexpressed with *1810019D21RIK*, *GM40444*, *GM31718* and *MYPOPOS* (see Fig. 6). It signified that both *IL10* and *FOXP1* acted as the hub target genes, and their immunoregulatory functions were probably mediated by different lncRNAs.

According to the reported biological functions of *RUNX1* and *IL4RA*, both of them play vital roles in the pathogenesis of AIH [56, 57]. An increased expression of *RUNX1* and *IL4RA* was discovered in our study as well, and there were four and nine DELs coexpressed with *RUNX1* and *IL4RA* at the threshold of |PCC| ≥ 0.99, although these DELs have not yet been validated with qRT‐PCR assay. The results presented here gave us some hints about how to explore the regulatory mechanism on *RUNX1* and *IL 4RA* in‐depth.

## Conclusions

In summary, a genome‐wide overview of aberrantly expressed lncRNAs and mRNAs in the Con A‐induced AIH mouse model was presented for the first time in our research. These DEMs and DELs have the potential to be the novel biomarkers for diagnosing and treating AIH (especially for these eight DELs and eight DEMs validated with qRT‐PCR). We also preliminarily determined the biological function of these screened DELs and DEMs, and the DEL‐DEM coexpressed networks were also constructed, which have provided some clues to further explore the effects of our identified DELs in the pathogenesis of AIH.

## Conflict of interest

The authors declare no conflict of interest.

## Author contributions

YL analyzed and interpreted the data, wrote the original draft. HC, J‐hH and Z‐cL acquired the data. TH and H‐qH conceived and designed the project, reviewed and edited the paper.

## Supporting information


**Table S1.** Top 10 up‐ and down‐regulated DELs with the descending order of |FC|.
**Table S2.** Top 10 up‐ and down‐regulated DEMs with the descending order of |FC|.
**Table S3.** Top 20 DELs‐DEMs coexpression pairs with the ascending order of *P* value.
**Table S4.** Top 3 negatively and positively expressed DMEs of each validated DEL with the descending order of |PCC| and the features of coexpression relationship.

## Data Availability

All the raw data are available from the corresponding author upon reasonable request.
