# Supplementary material for: Microarray‐based transcriptional profiling of a mouse model of autoimmune hepatitis
Source: FEBS Open Bio. 2020 Sep 19;10(10):2040–54. doi: 10.1002/2211-5463.12953 (PMC7530384; doi:10.1002/2211-5463.12953)
Supplement: Supplementary file 1 — Table S1. Top 10 up‐ and down‐regulated DELs with the descending order of |FC|. Table S2. Top 10 up‐ and down‐regulated DEMs with the descending order of |FC|. Table S3. Top 20 DELs‐DEMs coexpression pairs with the ascending order of P value. Table S4. Top 3 negatively and positively expressed DMEs of each validated DEL with the descending order of |PCC| and the features of coexpression relationship. [file FEB4-10-2040-s001.pdf]

Supplementary Table 1. Top 10 up- and down-regulated DELs with the descending order of |FC|

Accession	Genesymbol	*P*-value	Fold change	Regulation
XR_879185.1	Gm39247	8.27e-06	91.05	up
NR_033450.1	Serpina3h	9.24e-07	47.22	up
NR_038116.1	Cxcl11	7.54e-05	31.25	up
XR_878665.1	LOC102634900	3.14e-04	30.53	up
NR_132129.1	Gbp11	3.61e-07	28.13	up
XR_001782679.1	Gm36043	1.86e-05	25.80	up
XR_878666.1	OC102634900	2.64e-06	24.27	up
XR_373489.3	Gm30308	9.37e-03	19.23	up
XR_877803.2	Gm36043	1.61e-04	17.86	up
XR_378536.2	Gm34365	3.18e-04	17.74	up
XR_878680.1	GM41999	1.95e-06	-42.36	down
XR_878679.1	GM41999	3.36e-06	-39.31	down
XR_874527.1	GM31131	1.17e-05	-27.85	down
XR_878681.2	GM41999	1.39e-04	-26.24	down
XR_390613.2	GM21286	2.08e-05	-24.54	down
XR_877812.1	GM36419	1.92e-05	-21.75	down
XR_001781220.1	GM31131	7.45e-06	-21.52	down
XR_383357.2	GM31131	2.57e-04	-19.87	down
XM_011250153.2	MUP-PS16	4.45e-04	-16.05	down
XM_011250156.2	MUP-PS16	5.55e-04	-15.86	down

Supplementary Table 2. Top 10 up- and down-regulated DEMs with the descending order of |FC|

Accession	Gene symbol	*P*-value	Fold change	Regulation
XR_001785081.1	Olr1	1.39e-09	615.59	up
NM_008871.2	Serpine1	5.56e-12	606.56	up
NM_011315.3	Saa3	1.73e-07	555.30	up
NM_175628.3	A2m	1.56e-05	522.78	up
NM_001301094.1	Olr1	3.69e-09	481.41	up
XM_017321300.1	Olr1	8.30e-10	468.07	up
NM_008599.4	Cxcl9	2.11e-07	462.95	up
NM_001301096.1	Olr1	3.41e-08	443.99	up
NM_011330.3	Ccl11	7.52e-06	430.95	up
NM_138648.2	Olr1	7.44e-08	400.66	up
NM_007824.2	Cyp7a1	5.70e-04	-244.73	down
XM_006537604.2	Cyp7a1	2.68e-04	-111.48	down
XM_006537603.1	Cyp7a1	4.18e-04	-108.20	down
NM_008086.2	Gas1	5.93e-04	-41.92	down
XM_017313463.1	Usp2	2.35e-06	-33.03	down
XM_006518665.3	Lect1	1.43e-04	-28.81	down
NM_010701.3	Lect1	1.54e-04	-28.74	down
XM_006530657.3	Ces3b	1.03e-06	-25.58	down
XM_006524578.1	Tmem204	5.70e-05	-25.30	down
XM_006501729.3	Smad9	5.80e-07	-24.99	down

Supplementary Table 3. Top 20 DEL-DEM co-eression pairs with the ascending order of *P*-value

LncRNA Accession	LncRNA Symbol	mRNA Accession	Gene symbol	Correlation coefficient	*P*-value
XR_879821.1	GM42074	NM_001082552.2	TRIM21	0.999736712	4.56e-11
XR_001782924.1	LOC108168645	XM_006520578.3	SLC11A2	0.999639794	1.17e-10
XR_881095.1	GM33539	NM_011862.3	PACSIN2	-0.999625997	1.31e-10
XR_874481.1	GM35164	XM_006532716.2	SREBF1	0.999538999	2.45e-10
XR_878666.1	LOC102634900	XM_006539980.2	TARM1	0.999503873	3.05e-10
XR_871273.1	GM40579	NM_021704.3	CXCL12	0.999484476	3.42e-10
XR_872114.1	GM40781	XM_006527999.3	STARD8	-0.999478895	3.54e-10
NR_130109.1	FENDRR	NM_176835.2	DNAJC22	0.999457612	3.99e-10
XR_866821.2	9030622O22RIK	NM_019830.3	PRMT1	-0.999401433	5.36e-10
XR_872804.1	GM40438	NM_029880.3	PTGR2	0.999397854	5.46e-10
XR_375276.3	GM32287	NM_001033767.3	GM4951	0.999350462	6.85e-10
XR_871774.2	1110002J07RIK	XM_006495719.2	IL1R1	0.999331009	7.48e-10
XR_001781019.1	GM41018	NM_177192.3	DENND5B	-0.999301806	8.50e-10
NR_033450.1	SERPINA3H	NM_001033335.3	SERPINA3F	0.999266756	9.85e-10
XR_876895.1	GM34038	NM_001081239.2	LILRA5	-0.999259896	1.01e-09
XR_373577.2	GM33970	NM_146942.1	OLFR351	0.999241599	1.09e-09
XR_106464.4	GM20036	NM_018746.4	ITIH4	0.999227815	1.15e-09
XR_867009.1	GM32845	XM_011242602.1	TMEM25	-0.999217756	1.20e-09
XR_001784890.1	GM36764	NM_001252476.1	PRMT1	0.999204439	1.26e-09
XR_001779788.1	1110002J07RIK	NM_145512.4	SFT2D2	-0.999199355	1.28e-09

Supplementary Table 4. Top 3 negatively and positively eressed DMEs of each validated DELs with the descending order of | PCC value | and the features of co-eression relationship

LncRNA Accession	LncRNA Symbol	mRNA Accession	Gene Symbol	Correlation Coefficient	*P*-value
XR_001782679.1	GM36043	XM_006539980.2	TARM1	0.997094507	6.12e-08
		XM_017319372.1	LOC108168756	0.996459939	1.11e-07
		NM_030704.3	HSPB8	0.99547711	2.31e-07
		NM_010570.4	IRS1	-0.998920484	3.14e-09
		XR_875291.2	RAPGEF3	-0.997109801	6.02e-08
		XM_006533669.2	ARHGEF15	-0.995048509	3.02e-07
NR_137283.1	G530011O06RIK	XM_006526705.2	IFIT2	0.999089684	1.88e-09
		XM_017319260.1	FMNL2	0.999045188	2.17e-09
		XM_006533848.2	TNIP1	0.998905868	3.27e-09
		XM_006528006.1	STARD8	-0.998808335	4.23e-09
		NM_001313757.1	ACVR2B	-0.998561499	7.43e-09
		XM_006506025.3	ANKRD26	-0.996866127	7.68e-08
XR_879368.2	GM31718	NM_001271456.1	TNIP1	0.998638653	6.30e-09
		XM_011250345.2	SPSB1	0.998137905	1.61e-08
		NM_201389.2	PLEC	0.998061897	1.82e-08
		NM_010286.4	TSC22D3	-0.997807493	2.63e-08
		NM_177784.4	KLHL23	-0.996342908	1.22e-07
		XR_380378.1	PIP5K1C	-0.995390217	2.44e-07
NR_033590.1	GM8096	XR_001785081.1	OLR1	0.99906648	2.03e-09
		XM_006516132.3	SERPINA3I	0.998991103	2.57e-09
		NM_008871.2	SERPINE1	0.998988264	2.59e-09
		XM_006499465.2	FAM171B	-0.998465442	9.02e-09
		NM_024473.3	BC005537	-0.998002149	1.99e-08
		XM_006526027.3	ARHGEF37	-0.997761963	2.80e-08

NR_040346.1	1810019D21RIK	XM_006539769.3	SUV420H2	0.996454226	1.11e-07
		XM_011249052.2	BAHCC1	0.996002913	1.59e-07
		XM_006511138.3	PXYLP1	0.995788715	1.86e-07
		XM_006495799.2	PTP4A1	-0.998544575	7.70e-09
		NM_007961.4	ETV6	-0.996955211	7.04e-08
		XM_006495798.3	PTP4A1	-0.996672	9.19e-08
XR_380679.2	GM32468	NM_178727.2	D630039A03RIK	0.986758135	5.75e-06
		NM_024289.2	OSBPL5	0.986608309	5.94e-06
		XM_006524501.3	RMDN2	0.98525926	7.92e-06
		NM_001162489.1	MROH1	-0.979308841	2.18e-05
		XM_017321386.1	A530032D15RIK	-0.973764761	4.43e-05
		XM_006513314.3	KITL	-0.965879449	9.68e-05
XR_872832.2	GM40444	XM_017318104.1	SIPA1	0.996008212	1.59e-07
		XM_006526490.3	ZFP236	0.992470189	1.06e-06
		NM_153459.4	DUSP7	0.991039999	1.79e-06
		NM_175331.3	NT5DC3	-0.995230509	2.70e-07
		XM_017315015.1	NOL10	-0.99379176	5.95e-07
		NM_178608.4	REEP1	-0.992924708	8.81e-07
XR_001778054.1	MYPOPOS	XM_006539771.3	SUV420H2	0.997523534	3.79e-08
		NM_007634.4	CCNF	0.996881874	7.56e-08
		XM_006529624.3	COPS7B	0.995553808	2.19e-07
		XM_011241208.2	ETV6	-0.997302406	4.90e-08
		XM_006506669.3	SHQ1	-0.996740951	8.63e-08
		XM_011242651.2	DENND4A	-0.996521947	1.05e-07
